# Primary Salivary Human Stem/Progenitor Cells Undergo Microenvironment‐Driven Acinar‐Like Differentiation in Hyaluronate Hydrogel Culture

**DOI:** 10.5966/sctm.2016-0083

**Published:** 2016-08-18

**Authors:** Padma Pradeepa Srinivasan, Vaishali N. Patel, Shuang Liu, Daniel A. Harrington, Matthew P. Hoffman, Xinqiao Jia, Robert L. Witt, Mary C. Farach‐Carson, Swati Pradhan‐Bhatt

**Affiliations:** ^1^ Department of Biological Sciences, University of Delaware, Newark, Delaware, USA; ^2^ Center for Translational Cancer Research, Helen F. Graham Cancer Center & Research Institute, Newark, Delaware, USA; ^3^ Matrix and Morphogenesis Section, National Institute of Dental and Craniofacial Research, NIH, Bethesda, Maryland, USA; ^4^ Department of Materials Sciences and Engineering, University of Delaware, Newark, Delaware, USA; ^5^ Department of BioSciences, Rice University, Houston, Texas, USA; ^6^ Department of Biomedical Engineering, University of Delaware, Newark, Delaware, USA; ^7^ Department of Otolaryngology–Head & Neck Surgery, Thomas Jefferson University, Philadelphia, Pennsylvania, USA; ^8^ Department of Bioengineering, Rice University, Houston, Texas, USA

**Keywords:** Salivary glands, Stem/progenitor cells, Hyaluronic acid, Tissue engineering, Three‐dimensional hydrogel culture

## Abstract

Radiotherapy for head and neck cancer often has undesirable effects on salivary glands that lead to xerostomia or severe dry mouth, which can increase oral infections. Our goal is to engineer functional, three‐dimensional (3D) salivary gland neotissue for autologous implantation to provide permanent relief. An immediate need exists to obtain autologous adult progenitor cells as the use of embryonic and induced pluripotent stem cells potentially pose serious risks such as teratogenicity and immunogenic rejection. Here, we report an expandable population of primary salivary human stem/progenitor cells (hS/PCs) that can be reproducibly and scalably isolated and propagated from tissue biopsies. These cells have increased expression of progenitor markers (K5, K14, MYC, ETV4, ETV5) compared with differentiation markers of the parotid gland (acinar: MIST1/BHLHA15 and AMY1A; ductal: K19 and TFCP2L1). Isolated hS/PCs grown in suspension formed primary and secondary spheres and could be maintained in long‐term 3D hydrogel culture. When grown in a customized 3D modular hyaluronate‐based hydrogel system modified with bioactive basement membrane‐derived peptides, levels of progenitor markers, indices of proliferation, and viability of hS/PCs were enhanced. When appropriate microenvironmental cues were provided in a controlled manner in 3D, such as stimulation with β‐adrenergic and cholinergic agonists, hS/PCs differentiated into an acinar‐like lineage, needed for saliva production. We conclude that the stem/progenitor potential of adult hS/PCs isolated without antigenic sorting or clonal expansion in suspension, combined with their ability to differentiate into specialized salivary cell lineages in a human‐compatible culture system, makes them ideal for use in 3D bioengineered salivary gland applications. Stem Cells Translational Medicine *2017;6:110–120*

**Significance Statement**

Therapeutic irradiation for locally invasive head and neck cancers often leads to xerostomia or severe dry mouth, which greatly impairs quality of life. With current remedies being unsatisfactory, engineering an implantable salivary gland capable of restoring salivary functions of these patients is a promising solution. A major milestone toward salivary gland engineering is the identification and isolation of an expandable population of adult salivary human stem/progenitor cells (hS/PCs). Here we report a population of salivary hS/PCs that can be reliably isolated in scalable quantities, maintained long‐term in two‐dimensional/three‐dimensional (3D) culture, and used to produce a variety of salivary epithelial cell types for use in our human‐compatible, 3D engineered hydrogel model. Our ultimate goal is to use these cells to regenerate a functional autologous implantable salivary gland fully capable of reversing xerostomia.

## Introduction

Head and neck cancer is the fifth most common cancer in United States, accounting for 3%–5% of all malignancies. Despite improved protocols to prevent radiation scatter, radiotherapy for head and neck cancers often triggers apoptosis of the acinotubular compartment of the salivary glands resulting in chronic dry mouth, or xerostomia. The resulting loss of salivary function or hyposalivation can lead to difficulty swallowing, hampered speech, increased rate of oral infections, and reduced quality of life 1, 2. Currently available treatments including oral sialagogues and salivary stimulants offer short‐lived therapeutic benefit and are mainly palliative. Therefore, our goal is to bioengineer autologous salivary gland neotissue to restore salivary function for patients suffering from radiation‐induced xerostomia.

A major milestone in organ engineering involves the identification and isolation of stem/progenitor cells that can be differentiated into desired functional cell types by providing them with appropriate extracellular stimuli. The potential use of induced pluripotent stem cells (iPSCs) and bone marrow stem cells (BMSCs) has been evaluated for salivary gland restoration 3 4 5. Although iPSCs have the potential for regeneration, they pose undesirable risks that include genetic mutations, immunogenicity, and teratogenicity 6 7 8. The ability of BMSCs to transdifferentiate into salivary gland‐like cells via use of induction medium was recently evaluated 5. The use of mobilized BMSCs led to some functional recovery of damaged glands but much of it was attributed to surviving glandular stem/progenitor cells and growth factor stimulation 3, 9. The isolation of autologous salivary gland adult stem/progenitors reintroduced to the xerostomia patient can circumvent these challenges both by their lack of immunogenicity and by their potential to be differentiated into the desired cell type by providing the appropriate microenvironment.

Salivary glands contain diverse progenitor cell populations that give rise to the various cellular components in the salivary epithelium. Early work reported regeneration of the salivary gland following ligation‐induced acinar cell atrophy that was attributed to the presence of stem/progenitor cells in the ductal compartment 10, 11. Other groups reported that salivary gland‐derived stem/progenitor cells of mouse or human submandibular origin expanded using salisphere culture expressed markers such as Sca‐1, Kit, and Musashi‐1 12, 13. These sphere‐forming cells also were suggested to originate from salivary gland ducts with the ability to differentiate into amylase‐producing acinar‐like cells 12, 14.

Genetic lineage tracing in the developing mouse embryonic submandibular gland (SMG) indicates that progeny expressing the progenitor marker cytokeratin 5 (K5) are found throughout the adult glandular epithelium 15. It also was reported that K5 occurs in the proximal end buds in the developing mouse SMG and that cells expressing this marker can differentiate into a K19^+^ ductal lineage 16. In the adult mouse parotid gland and SMG, K5^+^, K14^+^, and Kit^+^ cells colocalized with EdU label‐retaining cells (LRC) in the acinar compartment, suggesting that LRCs comprise a heterogeneous population of progenitors 17. K14 was also present along with KIT in the distal end buds of the developing SMG 16. A recent report also suggests that K14^+^ ductal cells represent a population of stem cells that is established during development and contribute to the homeostasis of granular ducts in adult mouse SMG 18. Furthermore, KIT and FGFR2b signaling are important for the expansion and maintenance of the distal progenitors, and transplantation of Kit^+^ cells led to restoration of glandular function in mice 16, 19. It is, therefore, likely that several populations of progenitors exist in the murine salivary gland that can give rise to multiple glandular lineages and also that multiple progenitors may be needed for bioengineered tissue generation. Much less is known about the location and possible fates of the comparable cells in the human salivary glands.

Although stem/progenitor populations have been proposed to be involved in salivary gland homeostasis, it was recently reported that acinar cells also self‐duplicate to replace aged or injured cells 20, 21. However, radiotherapy depletes both these sources of self‐renewal, reiterating the need for isolating human stem/progenitor cells capable of giving rise to acini. It is unclear if this stem/progenitor cell population also must produce the other component cell types of the gland including ductal and myoepithelial cells, or whether resident surviving cells in the residual salivary gland tissue will integrate with the implant to provide these functions.

Although most of our current understanding about the salivary gland progenitors was gleaned from rodent models, there are a few reports on the isolation of human salivary gland‐derived stem/progenitor cells. Earlier work has demonstrated the isolation of adult stem cells expressing mesenchymal stem cell markers (CD49f, CD90, and CD105) or epithelial stem/progenitor markers (KIT, K5) and analyzed their potential to differentiate given the appropriate microenvironmental cues 13, 22 23 24 25 26 27 28. All of these studies use animal‐derived reagents (such as fetal bovine serum, type I rat tail collagen, or Matrigel), which cannot be translated to human systems. We previously reported the isolation of primary human salivary epithelial cells that can form functional lobular spheroid structures with large lumens when grown in unmodified hyaluronate (HA) three‐dimensional (3D) hydrogels 29 30 31. We also showed that salivary spheroids expressing the stem cell marker, CD44, could be maintained in implanted hydrogels for more than 1 week in our novel parotid gland resection model 32. Here we report a population of salivary human stem/progenitor cells (hS/PCs) that can be reliably isolated and expanded in sufficient numbers, without antigenic sorting or clonal expansion in suspension culture, suitable for use in a unique 3D hydrogel model of a human implantable salivary gland. We also report that our human‐compatible, peptide‐modified modular 3D HA culture system permits controlled differentiation to an acinar phenotype with luminal structures. The system is designed to be compatible with transplantation into humans. The isolation of hS/PCs and their controlled microenvironment‐driven differentiation into functional, self‐duplicating human acinar‐like cells (hSACs) represents a major stride toward engineering a human‐compatible salivary gland to restore salivary function in patients suffering from radiotherapy‐induced xerostomia.

## Materials and Methods

### Cell Culture

Human salivary gland tissues were procured from consented patients undergoing surgery for head and neck cancer following protocols approved by institutional review boards at both Christiana Care Health Systems and the University of Delaware. Explant culture for the isolation of hS/PCs was adapted from our previous isolation protocols 29, 31. Human parotid gland (hPG) tissue was disinfected with 1% (vol/vol) betadine solution in cold Dulbecco's Modified Eagle Medium (DMEM)/Nutrient Mixture F‐12 (Thermo Fisher Scientific, Grand Island, NY, https://www.thermofisher.com), minced into a slurry of small pieces and suspended in hepato‐STIM medium (Corning Inc. ‐ Life Sciences, Oneonta, NY, http://www.corning.com) supplemented with 1% (wt/vol) penicillin‐streptomycin, 1% (vol/vol) Fungizone (Thermo Fisher), and epidermal growth factor (EGF) (10 ng/ml). On reaching 70%–80% confluence, cells were trypsinized using 0.05% (wt/vol) trypsin‐EDTA (Thermo Fisher) that was stopped by adding trypsin soybean inhibitor (Sigma‐Aldrich, St‐Louis, MO, http://www.sigmaaldrich.com). Passages 2 through 15 were used for this study and all the experiments were repeated with cells isolated from at least 3 different patients.

### Unmodified and Peptide‐Modified HA 3D Hydrogel Systems

Salivary gland cells were encapsulated in 3D using the HA‐based HyStem (ESI BIO, Alameda, CA, https://www.esibio.com), composed of thiolated HA (HA‐SH) and poly(ethylene glycol) diacrylate (PEGDA), as described previously 31. Basement membrane‐derived peptides consisting of human sequences, including acrylated‐YIGSR (LM1, AC‐GGGYIGSR from laminin) and acrylated‐IKVAV (LM2, AC‐GGGIKVAV from laminin) and acrylated‐perlecan domain IV (PlnDIV peptide, AC‐GGGTWSKVGGHLRPGIVQSG, from perlecan), were produced following standard solid state peptide synthesis protocols and functionalized with reactive acrylate (AC) through the N‐terminus 33 34 35. Cells at a density of 1 × 10^5^ cells per 125 μl of hydrogel were mixed with HA‐SH alone or with an equal mixture of 3 peptides at a concentration of 20 μg/ml each. These peptides were incubated with HA‐SH for 15 minutes at 37°C. PEGDA then was added and mixed thoroughly before dispensing them into 12‐mm cell inserts (Millipore, Billerica, MA, http://www.emdmillipore.com). The mixture was incubated at 37°C for approximately 20 minutes to allow for complete gelation before adding culture medium around the insert and on top of the cell/gel construct. The medium was refreshed every 7 days. The peptide inclusion studies involving comparison of unmodified/peptide‐modified gels and the differentiation studies involving treatment with neurotransmitter agonists were performed with 3D cultures that were maintained until >75% of the spheroids reached approximately 50 μm, which, in average, ranged from 20 to 25 days. Comparison of spheroid size and number of cells per spheroid between the unmodified and peptide‐modified gels was achieved by measuring the diameter of hS/PC spheroids or the number of cells in each spheroid (by counting the nuclei) in at least three separate microscopy images for each group (unmodified and peptide modified; approximately 36 spheroids/group were measured) by two blinded scorers. The 3D hydrogel cultures used for performing experiments depicted in Figure 4 onward were grown with the three basement membrane‐derived peptides mentioned above (LM1, LM2, and PlnDIV peptide).

### Viability Assay

To compare the difference in growth/viability between the unmodified and peptide‐modified gels, the PrestoBlue Viability assay was performed. A working solution was prepared by mixing PrestoBlue Cell Viability Reagent (Thermo Fisher) with cell culture media at a 1:10 ratio and this solution then was added to spheroid‐laden unmodified and peptide‐modified gels. Gels were incubated at 37°C for 3 hours before measuring the absorbance at 570 and 600 nm. The corrected absorbance was calculated according to the manufacturer's instructions.

### Sphere Assay for “Stemness”

The isolated hS/PCs grown in two‐dimensional (2D) cultures were trypsinized for use in the sphere assay. 1 × 10^3^ cells were suspended in 200 μl of serum‐free stem cell medium (a gift from Dr. Modarai/Dr. Boman, Christiana Care Health Systems) made with DMEM/F12 (1:1) supplemented with 1× B27 (Thermo Fisher), 20 ng/ml EGF (Sigma‐Aldrich), and 10 ng/ml fibroblast growth factor 2 (Sigma‐Aldrich). The cells were incubated in a 96‐well ultra‐low attachment plates (Corning Life Sciences, Lowell, MA, https://www.corning.com) for 7 days. For secondary sphere formation, primary spheres were centrifuged at 3000 rpm for 4 minutes, incubated with 0.25% (wt/vol) trypsin/EDTA and strained with cell strainer to obtain a single cell suspension. The cells then were incubated with fresh medium at the same density as the primary spheres for an additional 7 days, as previously described by Kanwar et al. 36. Cells were imaged at each time point.

### Biomarker Assessment by Quantitative Polymerase Chain Reaction

To compare the steady state mRNA levels of selected genes, RNA was extracted either from human parotid gland tissue, hS/PCs grown in 2D, or those grown in 3D hydrogels using the RNAeasy Mini Kit (Qiagen, Valencia, CA, https://www.qiagen.com). DNAse treatment (Turbo DNAfree, Thermo Fisher) was performed and 1 μg of mRNA, as measured by the NanoDrop spectrophotometer (Thermo Fisher) was used for cDNA synthesis (cDNA Synthesis Kit, BioRad, Hercules, CA, http://www.bio-rad.com). The reaction mix was as follows: 12.5 μl of 2× SYBR Green mix, 5 μl forward and reverse primers (4 μM), 0.5 μl (approximately 25 ng) of cDNA, and 7.5 μl of water. Primer sequences for genes used in this study are listed in supplemental online Table 1. Primers for *BHLHA15* were obtained commercially (PPH08411A‐200; Qiagen). Quantitative polymerase chain reactions (qPCRs) were performed in duplicate for 40 cycles using the ABI 7300 PCR system (Thermo Fisher). The obtained C_T_ values were normalized to the housekeeping gene *GAPDH* and the fold changes were calculated using the ΔΔC_T_ method 37, 38.

### Immunostaining for Biomarkers

Cryosections of 8‐μm thickness obtained from human salivary gland tissue or 2D cells plated in 8‐well chamber slides (Lab‐Tek chamber slides; , Sigma Aldrich, St. Louis, MO; http://www.sigmaaldrich.com/ were fixed using ice cold methanol (or 4% (vol/vol) paraformaldehyde for MIST1/BHLHA15) for 10 minutes. After rehydration for 5 minutes, cells were permeabilized using 0.2% (vol/vol) Triton X‐100 for 15 minutes at room temperature. Bovine serum albumin 3% (wt/vol) in phosphate‐buffered saline (PBS) was used for blocking overnight at 4°C. Primary antibodies recognizing K5, K14, KIT, MYC, MIST1/BHLHA15, or α‐amylase were added for 1 hour and incubated on a rotating platform at 37°C. Detailed information about antibodies used in this manuscript is listed in supplemental online Table 3. After 3 consecutive washes with 1× PBS for 10 minutes each, secondary antibody conjugated to Alexa 488 or Alexa 568 fluorophores (Thermo Fisher) and Draq 5 (Biostatus, Leicestershire, United Kingdom, http://www.biostatus.com) were added and samples were incubated for 1 hour on a rotating platform at 37°C. After three 1× PBS washes, Fluoro Gel (Electron Microscopy Sciences, Hatfield, PA, https://www.emsdiasum.com) was added, and slides were cover‐slipped and stored at 4°C. Three‐dimensional hydrogels were stained similarly as described earlier and as reported previously 31. Whole gels were fixed and stained by prolonging each step of the previously listed 2D cell staining protocol by 50% to allow complete diffusion of liquids through the gels. Samples were imaged using either a Zeiss 510 or a 710 laser scanning confocal microscope (Carl Zeiss AG, Oberkochen, Germany, http://www.zeiss.com).

### Statistical Analysis

Statistical analyses were performed using one‐way analysis of variance (ANOVA) followed by the Tukey‐Kramer post hoc test 39. *p* < .05 (noted with an asterisk in all relevant figures) was regarded as statistically significant. All experiments were repeated with cells and tissue obtained from at least three different patient samples. Quantification of the number and size of the spheroids grown in unmodified and peptide‐modified gels were performed by two blinded scorers using the LSM image examiner software (Zeiss).

## Results

### Isolated Primary hS/PCs Are Enriched for Stem/Progenitor Markers Over Adult Parotid Gland Tissue

**Figure 1:**
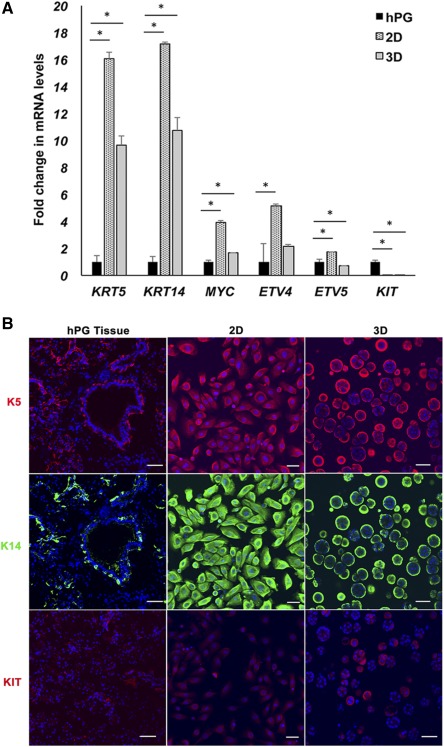
Expanded populations of human salivary gland cells display progenitor properties. **(A):** Expanded populations of human salivary gland cells cultured in 2D or 3D hyaluronate (HA) hydrogels are enriched for transcripts encoding progenitor markers when compared with whole adult hPG tissue. Error bars represent SEM. Statistical analysis was performed using one‐way analysis of variance followed by the Tukey‐Kramer post hoc test; ∗, *p* < .05. **(B):** Protein levels of stem/progenitor markers in hPG tissue, isolated human stem/progenitor cells (hS/PCs) in 2D and in 3D HA hydrogels. K14 (green) and K5 (red) staining is observed in a few cells within the ducts, along with myoepithelial cells in hPG tissue, and in the majority of the population of isolated hS/PCs in 2D as well as those cultured in 3D HA hydrogels. KIT staining (red) was seen in a relatively minor population in hPG tissue, 2D and 3D. Nuclei are stained in blue. Scale bars = 50 μm. Abbreviations: 2D, two‐dimensional; 3D, three‐dimensional; hPG, human parotid gland.

To evaluate the mRNA levels of stem/progenitor markers in primary cells freshly isolated from hPG tissue, qPCR was performed for previously reported stem/progenitor markers in salivary gland tissue (Fig. 1A). The marker genes chosen included K5 (*KRT5*) and K14 (*KRT14*), which are intermediate filaments; ETV4, ETV5, and MYC, transcription factors required for progenitor cell maintenance; and KIT, a progenitor marker and a receptor tyrosine kinase 40. Isolated cell populations displayed significantly higher mRNA levels encoding the progenitor markers *KRT5*, *KRT14*, *MYC*, and *ETV5* when compared with those of whole hPG tissue, except for KIT mRNA, which was significantly lower. This was true regardless of whether the cells were grown in 2D or encapsulated into unmodified 3D HA hydrogels. Levels of the transcripts encoding progenitor markers in 2D or 3D were not significantly different for *KRT5*, *KRT14*, and *ETV4*, regardless of culture condition. However, mRNA levels of *MYC* and *ETV5* were significantly higher in 2D when compared with 3D. KIT levels were significantly higher in 3D HA hydrogels when compared with 2D, although both are significantly lower compared with hPG.

To compare the protein staining intensity, subcellular and tissue localization of these stem/progenitor markers in adult hPG and isolated hS/PCs, hPG tissue sections and hS/PCs were stained with antibodies to K5, K14, and KIT (Fig. 1B). Both K5 and K14 were colocalized in a few interspersed cells within the parotid ducts and also were found in myoepithelial cells. Immunostaining of hS/PCs grown in 2D or 3D HA hydrogels revealed that K5 and K14 each were robustly expressed in a majority of the cells. KIT receptor, although present in hPG tissue, was seen at relatively low levels in small populations of cells in 2D and in 3D.

### Primary hS/PCs Retain Transcripts Encoding Stem/Progenitor Markers Over Multiple Passages and Can Be Maintained Long Term in 3D Hydrogel Culture

**Figure 2:**
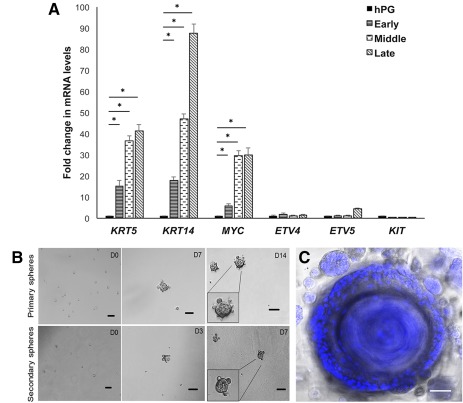
Primary human stem/progenitor cells (hS/PCs) retain transcripts encoding progenitor markers over multiple passages and retain stem/progenitor potential in long‐term culture. **(A):** hS/PCs retained transcript levels encoding stem/progenitor markers over passages 2 through 15 in two‐dimensional culture. Early: passages 2 and 3; middle: passages 9 and 10; late: passages 13 and 15. Error bars represent SEM. Statistical analysis was performed using one‐way analysis of variance followed by the Tukey‐Kramer test; ∗, *p* < .05. **(B):** Stem cell sphere forming assay. hS/PCs form primary and secondary spheroids when grown in stem cell media. Inset shows magnified image of spheroids. **(C):** A representative confocal image of an approximately 300‐μm three‐dimensional (3D) spheroid at its largest diameter, maintained for 118 days in 3D hyaluronate hydrogel culture. Nuclei are stained in blue. Scale bars = 50 μm. Abbreviations: D, day; hPG, human parotid gland.

To investigate the transcript levels of the selected stem/progenitor markers in hS/PCs with increasing number of passages, qPCR was performed on RNA isolated from the parotid cell populations cultured in 2D on plastic from passage 2 and 3 (early), passage 9 and 10 (middle), and passages 13 and 15 (late). Levels of stem/progenitor markers were maintained over several passages in hS/PCs grown in 2D (Fig. 2A). The transcripts encoding the stem/progenitor markers *KRT5*, *KRT14*, *MYC*, *ETV4*, and *ETV5* were increased in hS/PCs grown in 2D than in the hPG tissue irrespective of the passage number, suggesting enrichment and retention of a stem/progenitor pool ex vivo. To further support the stem/progenitor nature of the isolated hS/PCs, we performed the conventional sphere‐forming assay (Fig. 2B). Organized primary spheres seen by day 7 continued to grow and proliferate beyond day 14. Secondary sphere formation was observed as early as day 3 and continued to grow in size beyond day 7. Long‐term cultures of hS/PCs in 3D HA hydrogels retained their spheroid structures for more than 118 days (Fig. 2C).

### Incorporation of Bioactive Basement Membrane Peptide Sequences in 3D HA Hydrogels Enhances the Stem/Progenitor Properties of Cultured hS/PCs

**Figure 3:**
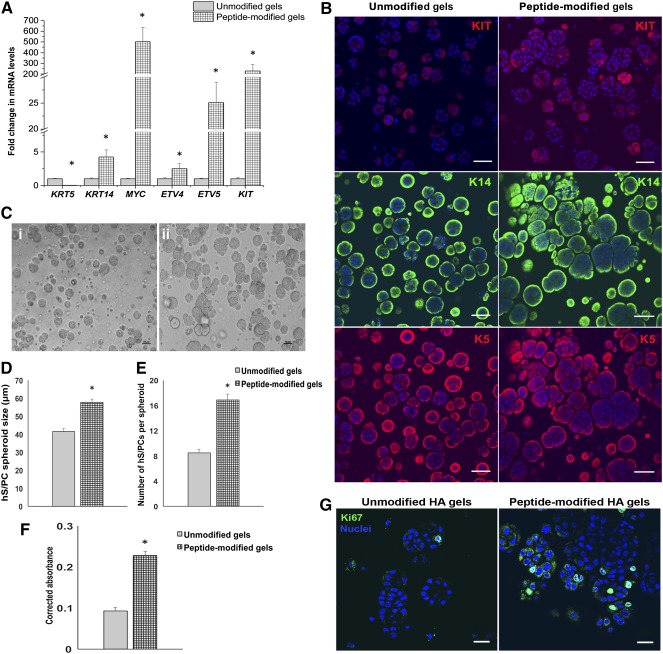
Bioactive basement membrane peptides enhance progenitor potential, viability, and proliferation of hS/PC spheroids in three‐dimensional (3D) HA hydrogels. **(A):** Basement membrane‐derived bioactive peptides from laminin (YIGSR, IKVAV) and perlecan (PlnDIV peptide) increase transcript levels encoding stem/progenitor markers in hS/PCs grown in 3D HA hydrogels. See study text for details. Error bars represent SEM. Statistical analysis was performed using one‐way analysis of variance; ∗, *p* < .05. **(B):** Spheroids cultured with basement membrane‐derived peptides gain expression of KIT in a majority of the population. Expression of K14 and K5 persists. Scale bar = 50 μm. **(C):** Brightfield image of hS/PC spheroids in unmodified 3D HA hydrogels **(Ci)** and in peptide‐modified HA hydrogels **(Cii)**. A significant increase in the size of spheroids **(D)** and number of hS/PCs per spheroid **(E)** is seen in HA hydrogels with basement membrane‐derived peptides. **(F):** hS/PC spheroids grown in peptide‐modified 3D HA gels display an increase in viability assessed via the PrestoBlue Assay. **(G):** Ki67 staining (green) indicates increased proliferation in hS/PC spheroids in hydrogels modified with basement membrane‐derived peptides. Nuclei are stained in blue. Scale bar = 20 μm. Abbreviations: HA, hyaluronate; hS/PC, human stem/progenitor cell.

Three bioactive peptides derived from two basement membrane proteins, laminin and perlecan, were each functionalized with a terminal acrylate group to enable their covalent attachment to the 3D HA hydrogel and to provide additional context cues to cells cultured in HA hydrogels. YIGSR and IKVAV (derived from laminin) and PlnDIV peptide (derived from domain IV of perlecan) were incorporated in the 3D HA hydrogel. We compared the stem/progenitor marker transcript levels, and the cell viability and proliferation, for hS/PCs grown in unmodified and peptide‐modified HA gels. We demonstrated a robust increase in overall mRNA yield and quality, suggesting the cells grow better in peptide‐modified HA gels ( supplemental online Table 2). There also were higher mRNA transcript levels encoding the progenitor markers, *KIT*, *MYC*, *KRT14*, *ETV4*, and *ETV5*, compared with those in the cells grown in unmodified HA gels (Fig. 3A). These 3D cultures (with and without peptide modification) were immunostained to assess protein content (Fig. 3B). Whereas K5 and K14 protein levels remained high in both conditions, a notable increase in the level of KIT protein, and in the number of cells expressing KIT, was observed for cells in 3D peptide‐modified HA gels (Fig. 3B). A significant increase in the overall size and number of cells per spheroid also was observed in the peptide‐modified HA hydrogels when compared with spheroids in unmodified gels (Fig. 3C, 3D, 3E). In addition, viability and proliferation of hS/PC spheroids was also increased with the presence of the three basement membrane peptides, as indicated by the PrestoBlue assay and Ki67 staining, respectively (Fig. 3F, 3G). Because the mRNA yield and stem/progenitor potential were consistently higher in hydrogels modified with basement membrane peptides, we used these same modified HA hydrogels for the rest of our studies.

### Primary hS/PCs Cultured in 2D or 3D Express Low Levels of Differentiated Marker Genes

**Figure 4:**
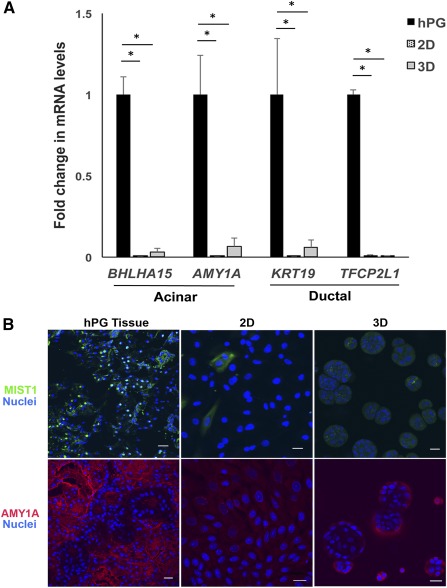
Transcript levels and localization of differentiation markers in tissue, 2D, and peptide‐modified 3D hyaluronate (HA) hydrogels. **(A):** Levels of acinar and ductal differentiation markers are significantly lower among human stem/progenitor cells grown in 2D and 3D peptide‐modified HA hydrogels compared with the whole hPG tissue. Error bars represent SEM. Statistical analysis was performed using one‐way analysis of variance followed by the Tukey‐Kramer test; ∗, *p* < .05. **(B):** Localization of MIST1/BHLHA15 and amylase in hPG tissue and 2D and 3D HA hydrogels with basement membrane‐derived peptides. Scale bar = 20 μm. Abbreviations: 2D, two‐dimensional; 3D, three‐dimensional; hPG, human parotid gland.

mRNA was isolated from hPG tissue and hS/PCs grown in 2D or 3D to compare the transcript levels of RNA encoding the acinar and ductal differentiation markers. In contrast to the stem/progenitor markers that were significantly higher in hS/PCs, mRNA levels of differentiation markers (acinar: *BHLHA15* and *AMY1A*; ductal: *TFCP21* and *KRT19*) were significantly lower in the hS/PCs (2D and 3D) when compared with those in hPG tissue (Fig. 4A). Similarly, other acinar‐related gene products such as *AQP5*, *PIP*, *PSP*, *HTN1*, *STATH*, and *MUC7* either were nondetectable or expressed at very low levels ( supplemental online Fig. 1). As expected, α‐amylase and MIST1/BHLHA15 proteins were present at high levels in the majority of cells observed in hPG tissue but were present at low levels, if at all, in hS/PCs grown in 2D or in 3D peptide‐modified HA hydrogels (Fig. 4B).

### Stimulation of hS/PCs Cultured in 3D HA Hydrogels Modified With Basement Membrane Peptides Increased Their Differentiation Toward an Acinar Lineage

**Figure 5:**
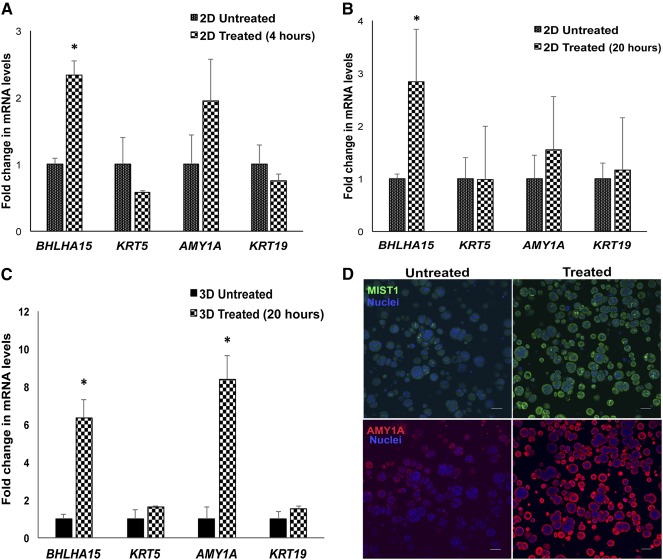
Stimulation of human stem/progenitor cells (hS/PCs) with β‐adrenergic and cholinergic agonists leads to differentiation of hS/PC spheroids toward an acinar lineage. Cotreatment with carbachol (25 μM) and isoproterenol (25 μM) increases levels of MIST1/BHLHA15 in hS/PCs cultured in 2D HA hydrogels at 4 hours **(A)** and 20 hours **(B)** after treatment. **(C):** Treatment with neurotransmitter agonists (25 μM carbachol and 25 μM isoproterenol) increases mRNA levels of acinar‐specific differentiation markers in hS/PCs cultured in 3D peptide‐modified hyaluronate hydrogels, 20 hours after treatment. Error bars represent SEM. Statistical analysis was performed using one‐way analysis of variance; ∗, *p* < .05. **(D):** Stimulation of hS/PCs in 3D with carbachol and isoproterenol increases acinar markers MIST1/BHLHA15 (green) and amylase (red) after 20 hours. Scale bar = 50 μm. Abbreviation: 2D, two‐dimensional; 3D, three‐dimensional.

Having evaluated the stem/progenitor phenotype of the hS/PCs, our next step involved differentiation of hS/PCs into α‐amylase secreting acinar cells. The ability to develop these secretory cells is a major building block necessary for creation of a fully functional engineered salivary gland model. hS/PCs were grown either in 2D or in 3D peptide‐modified HA hydrogels until a majority of the spheroids reached 50 μm in diameter (normally after 20–25 days in 3D culture) and were treated with the β‐adrenergic agonist, isoproterenol (ISO, 25 μM) and muscarinic agonist, carbachol (CCh; 25 μM) for 4 or 20 hours. qPCR was performed for acinar‐resident gene products *BHLHA15* (encoding the transcription factor, MIST1/BHLHA15) and *AMY1A* (encoding α‐amylase), ductal gene product *K19*, or progenitor specific gene product *K5* (Fig. 5A, 5B, 5C). Treatment with ISO and CCh significantly increased the levels of acinar gene products *BHLHA15* and *AMY1A* in agonist treated hS/PCs, studied both on 2D plastics and in 3D HA hydrogels. Two‐dimensional studies were performed first as a means for identifying the correct time‐points for agonist stimulation before undertaking the time‐dependent 3D experiments that necessitate prolonged culture for spheroid assembly. The increase in *BHLHA15* and *AMY1A* was noticeable within 4 hours of agonist treatment among hS/PCs grown in 2D (Fig. 5A). *BHLHA15* levels were further elevated at 20 hours after treatment, among 2D cells (Fig. 5B), making this an ideal time‐point for agonist treatment in 3D gels. Levels of *BHLHA15* and *AMY1A* were higher among spheroids in 3D gels than among 2D cells, 20 hours after treatment (Fig. 5C). The treatment did not significantly alter the levels of stem/progenitor or ductal markers. To confirm these findings at the protein level, the cells in the 3D gels were fixed and stained for MIST1/BHLHA15 and α‐amylase. Agonist stimulated cells in 3D peptide‐modified HA gels showed increased acinar markers BHLHA15 and AMY1A compared with the cells in untreated gels (Fig. 5D).

## Discussion

Expanded populations of human salivary stem/progenitor cells isolated from healthy tissue before radiotherapy potentially will be used to generate a bioengineered functional salivary gland replacement for patients suffering from xerostomia. This work reports the isolation of an hPG‐derived stem/progenitor population we call hS/PCs that can be differentiated into other salivary epithelial cell types. We define conditions for microenvironment‐driven differentiation of hS/PCs into amylase‐producing acinar cells suitable for incorporation in a 3D platform for autologous, postradiotherapy implantation.

To do this, we developed an explant culture protocol that promotes selective and reliable expansion of large quantities of hS/PCs from primary hPG tissue biopsies. The isolated cell populations possess significantly higher stem/progenitor markers than the native hPG tissue both in 2D and 3D HA hydrogel cultures. The formation of primary and secondary spheres by hS/PCs suggests the presence of a mixed stem/progenitor population and reflects the regenerative potential of isolated hS/PCs. Stem/progenitor markers in hS/PCs in 2D were maintained over several passages with no loss in expression. In addition, encapsulated cells in 3D can be maintained for more than 100 days. This stem/progenitor population also has the potential to be directed to differentiate into multiple cell types to recreate the complex salivary cell assemblies found in vivo.

The expanded stem/progenitor population retains many markers found in the basal/luminal progenitors of the duct. This finding is similar to those reported using salisphere cultures and in murine embryonic development 16, 41. We also showed that our cultures have cell‐surface localization of CD44, a stem cell marker and an HA receptor 32. We speculate that we have isolated multiple resident stem/progenitor populations from the intercalated duct, excretory ducts, and the acinar compartment. This mixed population may give rise to a variety of epithelial lineages of the salivary gland after injury if properly reactivated or if isolated, expanded and restored to the patient postradiation. This population may also include the ones proposed recently to “stream” from the intercalated duct to produce both acinar and ductal cells 18, 21.

Although mRNA levels of KIT were lower in hS/PCs relative to tissue, KIT was expressed by the hS/PCs in 2D and 3D. van Luijk et al. recently reported that expression levels of KIT could vary depending on the region of tissue biopsy because KIT^+^ cells were mainly concentrated in the central regions of the parotid gland surrounding the major ducts 42. In addition, KIT is highly expressed on some hematopoietic cells that may be within the gland at the time of biopsy. It is possible that selecting a specific KIT^+^ region of the gland to biopsy may improve autologous transplantation.

Jang et al. recently reported isolation of primary human minor salivary gland epithelial cells that expressed K5 and NANOG along with the differentiation markers AQP5, NKCC1, SLC12A2, K19, and vimentin 24. In contrast, our hS/PCs expressed low or nondetectable levels of differentiation markers and significantly higher levels of progenitor markers. Lombaert et al. also reported low levels of the acinar markers in unstimulated stem/progenitor cells in mouse salisphere culture 12. Uniquely, our hS/PCs retained low levels of differentiation markers and consistently higher stem/progenitor markers even after long‐term culture in 2D or in 3D HA hydrogels. Thus, our explant culture protocol allows expansion of hS/PCs with minimal differentiation, making them ideal candidates for controlled differentiation into specific salivary lineages. The ability of hS/PC spheroids to survive and proliferate for long periods of up to several months makes them ideal for use in an autograft model that requires cells to remain viable until the conclusion of treatment. Ongoing work will define other microenvironmental cues to drive the differentiation of all cell types needed to repair the gland.

To provide cues from the native microenvironment, bioactive basement membrane peptides from laminin and perlecan were incorporated into our 3D HA hydrogel system 43. We reported that PlnDIV peptide supports self‐assembly of acini‐like structures 29, 30. Here, we found that the incorporation multiple bioactive peptides in our 3D HA gels improved the stem/progenitor potential of hS/PCs with robust increases in *KIT*, *MYC*, and proliferation (Ki67 staining) and reduced *KRT5* expression, indicating a shift from a ductal toward a proliferating distal endbud progenitor state 16. It has been shown that KIT expression improves salivary gland morphology 12 and function in the radiated mouse salivary bed, and MYC is essential for stem cell maintenance and cell cycle progression in salivary tissue 40, 44. Thus, incorporation of multiple bioactive peptides into our 3D HA culture system improves expansion of the hS/PCs.

To recreate functional secretory structures and to evaluate the plasticity of our hS/PCs, we performed proof‐of‐concept studies designed to differentiate hS/PCs into secretory acinar cells. Neurotransmitter agonist stimulation directs progenitor cells into specific lineages 45, 46. We showed that treatment with β‐adrenergic and cholinergic agonists increased acinar markers such as MIST1/BHLHA15 and α‐amylase/AMY1A at both the gene and protein levels. The enhanced levels of α‐amylase in stimulated spheroids supports our previous work showing granule formation and secretion of α‐amylase upon neurotransmitter agonist stimulation 31. Similar to previous reports using mouse salispheres 47, MIST1/BHLHA15 protein was mainly observed in the periphery of the hS/PC spheroids and not in the nucleus. It is likely that complete differentiation of hS/PCs into secretory acinar cells will require coassembly or signals from other cell types, particularly the myoepithelial cells and endothelial cells.

## Conclusion

We report a reproducible ex vivo culture method to generate scalable quantities of human salivary stem/progenitor cells with stable expression of progenitor markers without antigenic sorting. The human stem/progenitor cells reported here can be maintained long‐term, in 2D as well as in 3D culture, without loss of biomarkers. The incorporation of bioactive basement membrane‐derived peptides in our 3D HA hydrogel enhances progenitor expansion. Upon stimulation with specific neurotransmitter agonists, these hS/PCs differentiate into specialized salivary acinar‐like cells. The isolation and directed differentiation of hS/PCs represents a major stride toward engineering a human‐compatible gland with potential to restore salivary function in patients suffering from xerostomia.

## Author Contributions

P.P.S.: collection and/or assembly of data, data analysis and interpretation, manuscript writing; V.N.P.: provision of study material, collection and/or assembly of data, data analysis; S.L.: provision of study material, data collection; D.A.H.: data analysis and interpretation, manuscript editing; M.P.H.: data interpretation, study design, provision of study material, manuscript editing; X.J.: data interpretation, provision of study material, financial support, manuscript editing; R.L.W.: provision of study material including institutional review board‐approved tissue biopsies, financial support; M.C.F.‐C.: conception and design, data interpretation, financial support, manuscript editing, final approval of manuscript; S.P.‐B.: conception and design, data analysis and interpretation, collection and/or assembly of data, manuscript writing, final approval of manuscript.

## Disclosure of Potential Conflicts of Interest

The authors indicated no potential conflicts of interest.

## Supporting information

Supporting Information
