# Supplementary material for: Primary Salivary Human Stem/Progenitor Cells Undergo Microenvironment‐Driven Acinar‐Like Differentiation in Hyaluronate Hydrogel Culture
Source: Stem Cells Transl Med. 2016 Aug 18;6(1):110–20. doi: 10.5966/sctm.2016-0083 (PMC5442728; doi:10.5966/sctm.2016-0083)
Supplement: Supplementary file 1 — Supporting Information [file SCT3-6-110-s001.pdf]

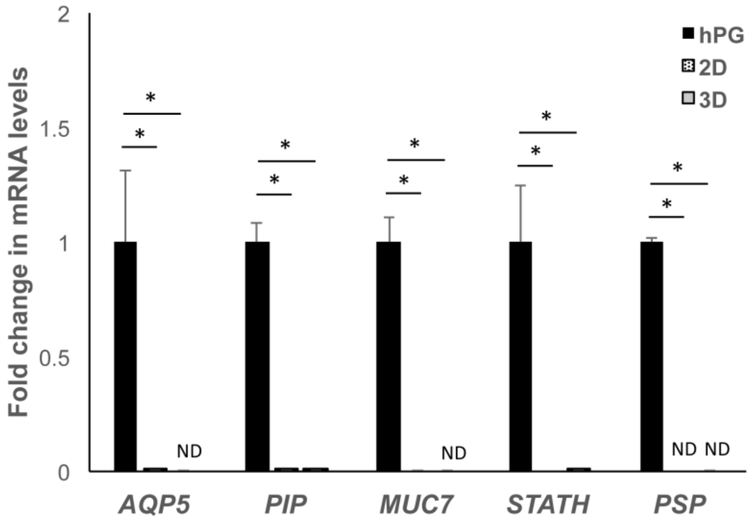

Figure S1. Levels of acinar differentiation markers are significantly lower among hS/PCs grown in 2D and 3D HA hydrogels compared to the native hPG tissue.

Supplemental Tables

Table S1. A list of primer sequences used in this study. Primers for *BHLHA15* were obtained commercially (Qiagen, NM_177455).

Table S1

Genes	Sense	Antisense
GAPDH	CAGCCTCAAGATCATCAGCA	TGTGGTCATGAGTCCTTCCA
RPS29	CAATATGTGCCGCCAGTGT	GAAGGAAGAGCATTTAGTCCAACT
K5	CGTGCCGCAGTTCTATATTCT	ACTTTGGGTTCTCGTGTCAG
K14	CACAGATCCCACTGGAAGAT	GATAATGAAGCTGTATTGATTGCC
MYC	CGGAACTCTTGTGCGTAAGG	TCATAGGTGATTGCTCAGGACAT
ETV4	CAGACGGACTTCGCCTAC	GGAATGGTCGCAGAGGTT
ETV5	GTGATACTGACAGGTAAGCAACA	AGAGCAGGCAGCAAGGTA
KIT	GTCTCCACCATCCATCCA	TCCATTCATTCTGCTTATTCTCA
AMY1A	CTCGGCACAGTTATTCGCAAGTGG	ACAGCCTAGCATCCCAGAAGGT
K19	CTGCCTCCAAGGTCCTCT	CCCATCCCTCTACCCAGAAG
TFCP2L1	GCCGCCTGCTTCCTGTTC	CTGCCCACCACTGCTCAAAG
PSP	TCTGAAGAGGACGAATGAGGAGGA	AGGCTTTCACGCTGGTTAGGT
STATH	AAGAGAACCCAGCCAACTATGAAG	TGTTGGTATTGTGGTTGGTATGGT
PIP	TCTCTGGGACACATTGCCTTCTG	TGATCTTCCGAGTGTTGTCCTGAG
AQP5	CTGTCCATTGGCCTGTCTGTC	GGCTCATACGTGCCTTTGATC
MUC7	GCTCCACCAGAGACCACAG	GCTGAGGCGGATGATGGG

Table S2. hS/PCs encapsulated in 3D HA hydrogels modified with basement membrane-derived peptides yield higher quality and quantity of mRNA than unmodified HA gels.

TABLE S2

Sample #	hS/PCs in Unmodified Gels		hS/PCs in Peptide-Modified Gels	
	mRNA (ng/µl)	260/280	mRNA (ng/µl)	260/280
1	9.4	1.74	25.9	1.72
2	13.1	1.43	31.3	1.78
3	4.8	1.47	23.7	1.71
Average	9.1	1.55	26.97	1.74

Table S3. A list of primary antibodies used in this study.

TABLE S3

Target protein	Vendor	Item #	Host Species	Dilution
**Cytokeratin 5 (K5)**	Biolegend	PRB160-P	Rabbit	1:1000
**Cytokeratin 14 (K14)**	Abcam	Ab49747	Mouse	1:100
**KIT**	Santa Cruz Biotechnology	SC1491	Goat	1:10
**Ki67**	BD Pharmingen	550609	Mouse	1:50
**MIST1**	Santa Cruz Biotechnology	SC80984	Mouse	1:50
**Amylase**	Sigma	A8273	Rabbit	1:100
